# Current Management Guidelines on Hyperlipidemia: The Silent Killer

**DOI:** 10.1155/2021/9883352

**Published:** 2021-07-31

**Authors:** Lilly Su, Rea Mittal, Devyani Ramgobin, Rahul Jain, Rohit Jain

**Affiliations:** ^1^School of Medicine, Pennsylvania State College of Medicine, Hershey, PA, USA; ^2^Touro College of Osteopathic Medicine, Middletown, NY, USA; ^3^Indiana University School of Medicine, Indianapolis, IN, USA; ^4^Department of Internal Medicine, Penn State Milton S. Hershey Medical Center, Hershey, PA, USA

## Abstract

Given the high incidence of cardiovascular events in the United States, strict control of modifiable risk factors is important. Pharmacotherapy is helpful in maintaining control of modifiable risk factors such as elevated lipids or hypercholesterolemia. Hypercholesterolemia can lead to atherosclerotic disease which may increase the risk of acute coronary events. Statin therapy has long been a mainstay in the treatment of hypercholesterolemia, but while highly regarded, statin therapy also has side effects that may lead to patient noncompliance. Therefore, various medicines are being developed to manage hypercholesterolemia. This paper will discuss the role that lipids play in the pathophysiology of atherosclerotic disease, review the current lipid management guidelines, and discuss new treatment options that are alternatives to statin therapy.

## 1. Introduction

Cardiovascular disease is a leading cause of mortality in America, claiming 65,000 lives annually [1]. Cardiovascular disease has been linked to many modifiable risk factors in the literature, particularly blood pressure, low density lipoprotein (LDL), high density lipoprotein (HDL), glucose intolerance, and smoking [2].

High total cholesterol, involving LDL, HDL, and triglycerides, is considered to be greater than or equal to 240 mg/dL. From 2015 to 2016, the CDC reported that 29 million (12.4%) of American adults had an elevated total cholesterol. Low HDL levels are considered less than 40 mg/dL of serum HDL. 18.4% of American adults had low HDL levels [3]. With a target LDL level of less than 100 mg/dL, 71 million (33.5%) U.S. adults had an elevated LDL level with only 23 million (33%) of these individuals having their LDL levels controlled [4].

It is increasingly important to control elevated cholesterol levels in American adults in order to decrease the prevalence of cardiovascular disease and the incidence of coronary events. In this paper, we will examine the 2018 American College of Cardiology/American Heart Association (ACC/AHA) guidelines. New drugs that are being developed to lower cholesterol since 2018 will be discussed.

## 2. Pathophysiology

Dyslipidemias involve clinically increased levels of cholesterol and/or triglycerides that may be accompanied with decreased HDL levels. Many of these patients have excessive hepatic VLDL production associated with elevated levels of triglycerides (TG), decreased levels of HDL cholesterol, and variable increased levels in LDL cholesterol. Excessive hepatic VLDL production can be driven by genetic factors, high carbohydrate diets, excessive alcohol use, obesity, insulin resistance, and nephrotic syndrome [5]. LDL levels have been causally associated with the risk of atherosclerotic cardiovascular disease (ASCVD) through absolute magnitude and cumulative duration of LDL-C exposure [6]. There is no current evidence for an established protective role for HDL against atherosclerosis [7].

LDL and HDL are the primary lipoprotein transporters of endogenous cholesterol esters; however, they also carry lesser amounts of triglycerides, phospholipids, and fat soluble vitamins. The lipoprotein lipid outer layer contains apolipoproteins that have several functions, including serving as a ligand for receptors and cofactors for enzymes and providing a structural basis for lipoproteins. LDL apolipoproteins include ApoB-100 and HDL apolipoproteins include ApoA-I, ApoA-II, ApoA-IV, ApoA-V, ApoC-III, and ApoE. Lipoproteins themselves serve to transport dietary lipids and hepatic lipids throughout the body to tissues that require fatty acid energy or storage. For example, HDL serves to transport cholesterol from peripheral cells to the liver and intestine. HDL transports peripheral cholesterol to the liver through scavenger receptor class B1 and exchanges cholesterol esters with TG from LDL cells through the cholesteryl ester transfer protein. The cholesterol esters transferred to the LDL particles can then be cleared from circulation through hepatic LDL receptor-mediated endocytosis. The cholesterol can then be hydrolyzed and excreted into bile [5].

**Figure 1:**
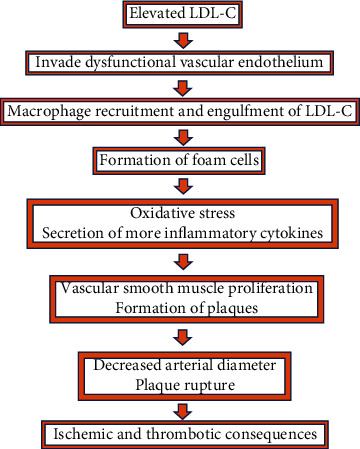
Pathophysiology of hypercholesterolemia leading to damage to the heart.

Elevated cholesterol in the body can lead to plaque formation and buildup within vasculature, leading to atherosclerotic cardiovascular disease as summarized in Figure 1 [8]. The three phases to the pathogenesis of atherosclerosis are initiation, progression, and complications. LDL cholesterol plays a large role in the initiation phase [7].

LDL particles can invade through dysfunctional vascular endothelium and cause subsequent inflammation. The likelihood of LDL intimal retention is affected by the endothelial phenotype and serum LDL levels. Transcription factor Krupper-like Factor 2 (KLF2) has been implicated in atheroprotective hemodynamics. KLF2 promotes anti-inflammatory, antithrombotic, and atherogenic action at the endothelium through the MEK5/MEF2 signaling pathway, decreasing the susceptibility of the vascular endothelium to invasive LDL particles causing atherosclerosis. HMG CoA reductase inhibitors, which are widely used in treating hypercholesterolemia, have also been found to upregulate the KLF2 expression which may contribute to the protective cardiovascular effects of this drug class [9]. The likelihood of LDL invasion into dysfunctional endothelium also depends on the serum levels of LDL. When LDL-C is higher than 20-40 mg/dL, there is a dose-dependent increase in the probability of LDL intimal invasion and progression to atherosclerosis [6].

After the LDL particles invade the dysfunctional endothelium, the lipid molecules can become altered, oxidized, or glycosylated into modified LDL particles [10]. Subendothelial LDL particles can also bind to intimal proteoglycans to aggregate together and enter smooth muscle cells, leading to further lipid accumulation [7]. In response to the LDL invasion of the endothelium, monocytes infiltrate the subendothelial space [11]. The macrophages take up the modified LDL particles and become foam cells, termed due to their high cholesterol load. These foam cells cause oxidative stress and secrete cytokines involved in plaque growth, and their death can promote inflammation, leading to vascular smooth muscle proliferation and the formation of plaques [12]. Recent studies suggest that smooth muscle cell metaplasia leads to foam cells resembling macrophages. While monocyte-turned-macrophages make up a majority of foam cells, smooth muscle cell migration from the media to intima contributes to the expanding plaque [7]. The process of atherosclerosis plaque formation can lead to decreased arterial diameter and plaque rupture, potentially causing ischemic or thrombotic consequences.

Lipid-lowering drugs have proven to be effective in reducing the risk of atherosclerotic cardiovascular disease, highlighting the causal link between dyslipidemia and atherosclerosis. Statins, HMG-CoA reductase inhibitors, reduce plasma LDL-C levels. A meta-analysis demonstrated a dose-dependent reduction in the risk of major cardiovascular events proportional to the reduction in LDL-C from treatment [6]. Ezetimibe inhibits intestinal absorption of cholesterol, where ezetimibe therapy was associated with a proportional reduction in cardiovascular events. PCSK9 monoclonal antibodies increase LDL clearance from the blood and are associated with a decreased risk of cardiovascular events. Therefore, dyslipidemia treatments decreasing lipid levels can greatly impact the cardiovascular outcomes of these patients [6].

## 3. Clinical Presentation

Hypercholesterolemia often lacks overt symptoms but is a major risk factor for cardiovascular disease [3]. Very high cholesterol levels, usually seen in familial hypercholesterolemia, can manifest as xanthomas and corneal arcus. Complications from inadequately managed hypercholesterolemia include carotid artery disease, stroke, peripheral vascular disease, high blood pressure, and type two diabetes mellitus (T2DM) [13, 14].

Systemic diseases associated with influencing dyslipidemia include psoriasis, Crohn's disease, inflammatory bowel disease, chronic obstructive pulmonary disease, depression, chronic pain, and chronic kidney disease [14]. A 2017 observational study involving 7,641 Europeans over the age of 50 reported that 1,591 (20.8%) of the patients had high triglyceride or low HDL levels. These patients were also more likely to be obese, have type two diabetes mellitus, and exceed the recommended weekly limit of alcohol consumption. 55% of the patients with elevated TG and low HDL were not receiving any lipid management [15].

Complications from hypercholesterolemia can be managed via adequate lipid management. There are many guidelines regarding proper administration of lipid-lowering drugs that are being developed to help lower cholesterol since the 2018 lipid guidelines. In this paper, we will summarize the ACC/AHA 2018 guidelines and examine new drugs that have been developed since the release of the 2018 guidelines.

## 4. 2018 AHA/ACC Guidelines

Further updates were made to the 2013 lipid guidelines when the ACC/AHA came out with the 2018 Guideline on the Management of Blood Cholesterol. It recognizes that although there is no ideal target blood level for LDL-C, it is of utmost importance to keep those levels low, as adults with LDL-C levels at or below 100 mg/dL are less likely to have heart disease and stroke [16]. In addition to examining risk factors that were commonly associated with cardiovascular disease such as high cholesterol, high blood pressure, and smoking, the 2018 guidelines also recommend looking into risk-enhancing factors in older patients aged 40 to 75, which include family history and other health conditions [16]. By doing so, it provides a more personalized risk discussion for the patient. Another important addition to the 2018 guidelines is the use of coronary artery calcium (CAC) scoring that is utilized to reclassify risk patients at either borderline or intermediate risk, for whom the risk of statin treatment is uncertain [15].

Regarding treating high cholesterol, lifestyle modifications such as decreased intake of saturated fat and exercising at least 40 minutes 3 to 4 times a week are typically the first step in treatment, unless the patient has a very high risk of ASCVD [16].

Not only does the 2018 guidelines allow for more personalized care and more treatment options with the introduction of nonstatin therapies such as ezetimibe and bile acid sequestrants, but it also includes additional risk-assessment strategies to prevent unnecessary prescription of statin treatments.

## 5. New Medications since 2018

Since the 2018 lipid-lowering guidelines came out, there have been a multitude of new lipid-lowering drugs that are currently undergoing extensive experimental studies in the hopes of potentially being brought to the market in the near future.

Inclisiran may potentially be the first and only LDL-C-lowering siRNA treatment. It is intended to be injected subcutaneously by a healthcare professional with an initial dose, a second dose 3 months later, and then, after that, every six months. By increasing LDL-C receptor recycling and expression on the hepatocyte cell surface, Inclisiran thereby increases LDL-C uptake by hepatocytes and lowers LDL-C levels in the bloodstream [17]. Evinacumab inhibits angiopoietin-like 3 (ANGPTL3), which itself is an inhibitor of lipoprotein and endothelial lipase that is responsible for increasing the levels of triglycerides and other lipids. As ANGPTL3 loss of function mutations is normally associated with lower levels of LDL-C independent of the LDL receptor, Evinacumab's mechanism of action attempts to achieve similar results [18]. Vupanorsen (AKCEA-ANGPTL3) reduces the production of the ANGPTL3 protein, which ultimately leads to reduction in LDL-C levels. In addition to dose-dependent reduction in ANGPTl3 and LDL-C, Vupanorsen has also shown to reduce TG, non-HDL-C, and total cholesterol [19]. Gemcabene is able to enhance the clearance of VLDLs in plasma and inhibition of production of cholesterol and TGs in liver through its liver-directed downregulation of hepatic apolipoprotein C-III (apoC-III) [20]. ARO-ANG3 also inhibits ANGPTL3 and is designed to reduce triglycerides and decrease LDL-C in patients with mixed dyslipidemia [21]. Bempedoic acid inhibits ATP-citrate lyase, which is a component of the cholesterol synthesis pathway [22].

Table 1 details some of these new drugs, their mechanism of action, side effects, and efficacy.

## 6. Current Status of These Drugs

Last year, the FDA rejected Inclisiran, but Novartis has reapplied. Evinacumab and Bempedoic acid have been approved by the FDA to be used as an adjunct with other cholesterol-lowering drugs. Vupanorsen has successfully completed its phase 2 trial, whereas Gemcabene has been put on hold for its phase 2 trial as the FDA is waiting for rat carcinogenesis data on Gemcabene. Finally, as of January 2021, an IND for a phase 2 study of ARO-ANG3 was submitted to the FDA.

## 7. Conclusion

When educating patients on their cholesterol, it is often not the total cholesterol that is important to mention but the actual LDL level. Sometimes termed “bad cholesterol,” low density lipoprotein is a great indicator of a patient's lipid profile. Ideal LDL levels should be less than 100 mg/dL; however in those who have heart disease or diabetes, optimal levels should either be less than 70 mg/dL or be reduced by at least 50%. The use of statin therapy is a key player in achieving optimal LDL levels, especially important in four high risk groups: patients with a greater than 7.5% ASCVD risk, those with a history of familial hypercholesterolemia, patients with a known history of atherosclerotic disease, and patients with diabetes mellitus. High intensity statins as well as adherence to diet and lifestyle modifications have proven to be effective at preventing coronary events. However, with the introduction of newer medications, there is the possibility of replacing statins. Thus, while statins may reign as the wonder drug of cholesterol management, there may be new contenders for the throne.

## Figures and Tables

**Table 1:** Drugs developed since release of the 2018 ACC/AHA cholesterol guidelines.

Drugs	Mechanism of action	Side effects	Efficacy
Inclisiran [17]	Small interfering RNA targeting hepatic PCSK9 synthesis	Possible myalgia, headache, fatigue, back pain, hypertension, and dizziness	Durable and potent reduction in LDL-C of 51% when used in addition to other lipid-lowering therapies over 17 months of treatment
Evinacumab [18]	Monoclonal antibody that inhibits ANGPTL3	Nasopharyngitis, influenza-like illness, headache, rhinorrhea	At week 24, patients in the Evinacumab group had a 47.1% reduction from baseline in LDL-C levels, as compared to an increase of 1.9% in the placebo group
Vupanorsen [19]	Targets ANGPTL3 with an antisense oligonucleotide	Most common adverse effects were injection site reactions	Statistically significant dose-dependent reductions compared to placebo in ANGPTL3 (62%), VLDL (38%), total cholesterol (19%), and non-HDL (18%)
Gemcabene [20]	Downregulation of hepatic apoC-III mRNA expression and decrease of plasma apoC-III	No severe adverse effects were observed	Gemcabene 300 mg and 900 mg produced a mean percent change in LDL-C of −23.4 ± 4.7% (*P* = .005) and −27.7 ± 4.3% (*P* < .001), respectively, vs. −6.2 ± 4.3% for placebo
ARO-ANG3 [21]	siRNA directed against ANGPTL3 mRNA	Headache, respiratory tract infections, and local injection site reactions	Hypercholesterolemia patients on a stable LDL-C-lowering treatment saw a mean maximum reduction in ANGPTL3 of 79-88% and LDL-C of 39-42% after receiving the first dose
Bempedoic acid [22]	Small molecule inhibitor of ATP-citrate lyase	Small elevation in mean uric acid levels	Treatment with Bempedoic acid reduced LDL-C significantly more than placebo at week 12 (placebo-corrected change from baseline, −21.4% (95% CI −25.1% to −17.7%); *P* < 0.001)
